# Identifying Genomic Regions Targeted During Eggplant Domestication Using Transcriptome Data

**DOI:** 10.1093/jhered/esab035

**Published:** 2021-06-15

**Authors:** Anna M L Page, Mark A Chapman

**Affiliations:** Biological Sciences, University of Southampton, Southampton SO17 1BJ, UK

**Keywords:** domestication, eggplant, selection, Solanum melongena, transcriptomics

## Abstract

Identifying genes and traits that have diverged during domestication provides key information of importance for maintaining and even increasing yield and nutrients in existing crops. A “bottom-up” population genetics approach was used to identify signatures of selection across the eggplant genome, to better understand the process of domestication. RNA-seq data were obtained for 4 wild eggplants (*Solanum insanum* L.) and 16 domesticated eggplants (*S. melongena* L.) and mapped to the eggplant genome. Single-nucleotide polymorphism (SNPs) exhibiting signatures of selection in domesticates were identified as those exhibiting high *F*_ST_ between the 2 populations (evidence of significant divergence) and low π for the domesticated population (indicative of a selective sweep). Some of these regions appear to overlap with previously identified quantitative trait loci for domestication traits. Genes in regions of linkage disequilibrium surrounding these SNPs were searched against the *Arabidopsis thaliana* and tomato genomes to find orthologs. Subsequent gene ontology (GO) enrichment analysis identified over-representation of GO terms related to photosynthesis and response to the environment. This work reveals genomic changes involved in eggplant domestication and improvement, and how this compares to observed changes in the tomato genome, revealing shared chromosomal regions involved in the domestication of both species.

By studying domestication, the human population can better understand events of the past and arm themselves against an uncertain future by breeding crops that can meet the demands of climate change and a growing population (Godfray et al. 2010). Despite being the second most important solanaceous fruit crop after tomato (Knapp et al. 2013), eggplant is relatively understudied. Recent advancements, such as the development of a reference genome and backcross populations for breeding, make this an ideal time to study the domestication of eggplant (Chapman 2019).

During the process of domestication, eggplants have been exposed to strong, directional, human-mediated selection. This established a suite of domestication syndrome traits that distinguish domestic eggplants from the wild progenitor *Solanum insanum* L., for example a loss of spines, larger and nonbitter fruits, and more consistent flowering (Page, Daunay, et al. 2019). However, because landraces and wild relatives typically occupy the same range, gene flow and hybridization are common between wild and domesticated populations (Davidar et al. 2015; Page, Gibson, et al. 2019). The complicated demographics this produces can make it challenging to identify true wild and domesticated accessions based on phenotype alone. For example, Knapp et al. (2013) proposed a key to morphological characteristics for distinguishing ambiguous specimens; however, we recently showed that some phenotypically wild eggplants were in fact feral domesticates with admixed (wild and domesticated) genomes (Page, Gibson, et al. 2019). Analyses in eggplant aiming to identify variants associated with selection during domestication must therefore be accompanied by demographic inference such as a phylogeny to verify true wild and true domesticated accessions.

Previous work to identify the genomic regions underlying phenotypes in eggplants have largely taken a top-down approach, most often analyses of quantitative trait loci (QTL). These analyses have revealed loci linked to domestication traits using crosses between domestic and wild eggplant parents (Doganlar et al. 2002; Frary et al. 2014; Portis et al. 2014; Toppino et al. 2016; Miyatake et al. 2020), and loci controlling specific traits that vary between cultivars such as disease resistance (Barchi et al. 2018), and anthocyanin content (Barchi et al. 2012).

Domestication imposes a bottleneck on the crop, which increases the strength of genetic drift. This will lead to a genome-wide reduction in diversity. Regions of the genome which have been under selection will have lower diversity still, driving the selected allele and the genomic regions surrounding it toward fixation (Burke et al. 2007; Olsen and Wendel, 2013). The eggplant reference genome recently developed by Barchi et al. (2019) brings the opportunity to identify regions under selection using a bottom-up approach; therefore, we used this to scan the genome for signals of selection without making a priori choices about the phenotypes of interest (Ross-Ibarra et al. 2007).

Previously, we analyzed RNA-seq data on a gene-by-gene basis (Page, Gibson, et al. 2019), as there was no reference genome available to us. This means that outlier loci we identified based on patterns of nucleotide diversity could be influenced by loci in linkage disequilibrium (LD). We therefore chose to reanalyze this data mapping the RNA-seq reads to the genome to identify more clearly patterns of selection during domestication. Looking at overall patterns of selection on the genome, and how these compare to other Solanaceous crops also reveals more about the effects artificial selection has had on the genome, such as what proportion of the genome was under selection, and whether the same regions were targeted in related crops.

## Materials and Methods

### Existing Datasets, RNA Extraction, and Sequencing

The RNA-seq data from Page, Gibson, et al. (2019) were reanalyzed in this work. We used the phylogenetic analysis of a genotyping-by-sequencing data set of 95 domesticated eggplants and wild relative species sampled across their range (Page, Gibson, et al. 2019) to define true wild and domesticated eggplants. True wilds are defined as wild accessions sister to the domesticates in the phylogeny, whereas phenotypically wild accessions nested within the domesticated clade with admixed genomes are defined as feral and are excluded here.

Briefly, one fully expanded leaf from each accession (4 wild and 16 domesticated; Table 1) was frozen in liquid nitrogen, and RNA was extracted using a Qiagen RNeasy Plant Mini kit (Qiagen, UK), utilizing an on-column DNase step (Qiagen). Samples were sent to the Wellcome Trust Centre for Human Genetics (WTCHG, Oxford, UK) for quantification, quality checking, and subsequent library preparation using Illumina’s Stranded Truseq kit (Illumina, UK). Up to 12 libraries (individually barcoded) were sequenced per lane on Illumina Hiseq2000 for 101 cycles (paired end). Samples were then demultiplexed and bioinformatic analyses took on the University of Southampton Iridis4 supercomputer. All samples are available from the NCBI Sequence Read Archive (PRJNA526115).

**Table 1.** Samples from which RNA-seq data were used

Accession^a^	Population	SRA accession	*N* reads	Country of origin	Spines^b^	Fruit color^b^	Fruit shape^b^
MM12137	Domesticated	SRR8736626	19 623 124	India			
MM1712	Domesticated	SRR8736644	11 685 753	China	No	Purple	Elongated
MM1791	Domesticated	SRR8736653	15 531 766	Vietnam	Yes (calyx)	Purple	Round
Mey319	Domesticated	SRR8736652	26 896 589	China	No	Purple	Round
PI241594	Domesticated	SRR8736633	18 493 989	Taiwan			
Arum	Domesticated	SRR8736631	21 310 738	India			
MM0609	Domesticated	SRR8736630	12 326 452	India	No	Purple	Round
MM0673	Domesticated	SRR8736638	22 010 086	India			
MM10439	Domesticated	SRR8736627	23 224 957	Maldives			
S00255B	Domesticated	SRR8736636	22 806 997	India			
S00392	Domesticated	SRR8736637	22 688 619	India	No	Purple	Elongated
MM12391	Domesticated	SRR8736632	23 184 716	Malaysia	No	Purple	Round
MM12454	Domesticated	SRR8736635	23 079 041	Indonesia			
MM1290	Domesticated	SRR8736634	14 460 317	Philippines	No	Purple	Elongated
MM1547	Domesticated	SRR8736629	15 435 197	Malaysia	No		
PI470273	Domesticated	SRR8736628	22 634 687	Indonesia	No	Purple	Elongated
MM0669	Wild	SRR8736648	22 235 038	India	Yes	Green	Round
MM0675	Wild	SRR8736647	18 680 378	India	Yes	Green, variegated	Round
PI381155	Wild	SRR8736645	18 387 079	India			
MM0686	Wild	SRR8736646	24 282 614	Indonesia			

^a^Accessions names beginning “MM” are from INRA, “Meyer” indicates collection of R. Meyer (UCLA), “PI” are from the USDA, “TS” and “S” are from the AVRDC, “ARUM” indicates a landrace from Amishland seeds.

^b^Phenotypic information is given where available.

### Transcriptome Variant Calling

RNA-seq reads were trimmed and poor quality bases and short reads removed using Trimmomatic v. 0.32 (Bolger et al. 2014) as described previously (Page, Gibson, et al. 2019), and aligned to the eggplant genome (Barchi et al. 2019) using STAR (Dobin et al. 2012). A gff3 file of predicted genes from the eggplant genome was supplied when indexing the genome with STAR, which improved mapping quality by providing annotations for known splice junctions. After mapping, Picard 2.8.3 (Broad Institute 2019) and GATK (McKenna et al. 2010) were used to process the mapped reads following the recommendations of GATK best practices for calling variants in RNA-seq, that is, marking duplicates in Picard, using SplitNCigarReads in GATK to split reads into exon segments, and calling variants with HaplotypeCaller in GATK. Hard filtering was then applied to the callset. GATK best practices recommend filtering clusters of 3 or more single-nucleotide polymorphism (SNPs) within a 35-base window, Fisher strand values > 30, and quality by depth values < 2. BCFtools within SAMtools v. 0.1.19 (Li et al. 2009) was used to merge the vcf files of samples into 2 files (wild and domesticated), then further filtering was done to remove any SNPs with a minor allele frequency (MAF) of <0.1, and with coverage in the lowest 99th percentile.

### Population Genomic Statistics

The *Populations* program in Stacks v. 1.48 (Catchen et al. 2013), with the vcf files as input, was used to calculate AMOVA *F*_ST_ between the domesticated and wild populations, and π within the domesticated population. SNPs were not filtered for missing data because of the small sample size in the wild population, which we assume adds some noise to the estimates of *F*_ST_ and π. AMOVA *F*_ST_ in Stacks was derived from Weir (1996) and was calculated as the weighted average of the surrounding 450 kb (the default value in Stacks) of sequence for each SNP. A weighted average of π in the surrounding 450 kb of sequence was also calculated for the domesticated population. 10^6^ bootstrap replicates were performed and used to report *P* values, which were subsequently corrected using the Holm–Bonferroni sequential correction (Holm 1979), calculated using the p.adjust command in *R* 3.1.3 (R Core Team 2015) on a per chromosome basis. These were considered outlier SNPs for high *F*_ST_, low π, and their overlap.

To identify the region of the genome in LD with outlier loci, haplotype blocks containing SNPs in “strong LD” (sensu PLINK) were estimated from the filtered combined wild and domesticated data set using PLINK 1.9 (Purcell et al. 2007; Chang et al. 2015). The cumulative percentage of haplotype blocks was then plotted against size of haplotype blocks and used to identify a point at which the curve leveled off. This was used to define regions of interest around outlier loci, and from within these regions, genes were identified.

### Gene Ontology Analysis

A fasta file containing the sequences of all annotated genes in the regions putatively under selection was created from the eggplant genome sequence using gff3 files to identify genomic positions. This fasta file was used in a BLAST search against *Arabidopsis thaliana* CDS sequences (ver. TAIR10) and tomato (ver. ITAG2.4) using Bioedit (Hall 1999), retaining significant hits (*e*-value < e^−10^). These lists of genes were then used in a gene ontology (GO) enrichment analysis (Ashburner et al. 2000) with agriGo (Du et al. 2010) to identify GO terms that were over-represented in the candidate gene list. False discovery rate correction was applied to the *P*-values.

## Results

### Mapping and Calling SNPs

Between 11.7 and 26.9 M reads were sequenced from each accession, with an average of 19.9 M (± 0.9 M [SE]). Annotated coding genes make up 8.90% of the 1.2 Gb eggplant genome, and of the 27 139 annotated coding genes in the eggplant reference genome, 22 954 were present in the transcriptome of at least one accession. Following hard filtering on the SNPs called, the average number of SNPs per sample decreased by 10.73%, from 117 699 to 105 075. After merging samples based on population, further filtering on MAF and coverage retained 203 611 out of a possible 668 552 SNPs.

### Haplotype Block Analysis to Estimate the Average LD in the Eggplant Genome

**Figure 1.**
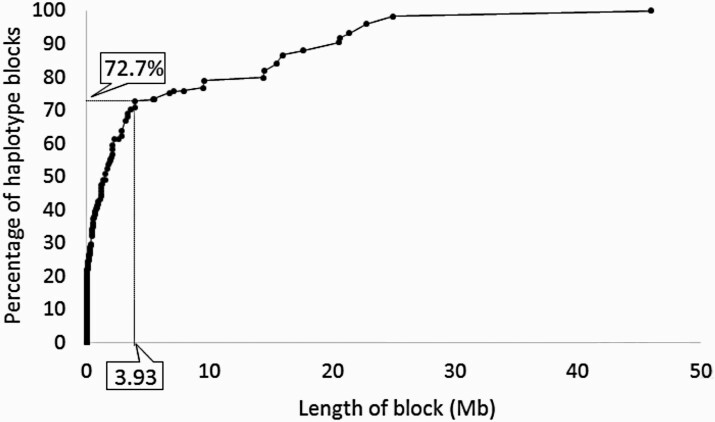
Cumulative percentage graph of haplotype block size. The point at which the rate of increase of cumulative percentage declines is marked on the x and y axes.

When the upper-class boundary of the estimated haplotype block sizes, estimated for the combined wild and domesticated data, was plotted against the cumulative percentage, there was a clear drop off in the rate of increase of haplotype block size (Figure 1). The majority of haplotype blocks (72.5%) are under 3.93 Mb in size, and following this point, increasing the length of block has diminishing returns for the increase in the cumulative percentage of haplotype blocks. Therefore, 3.93 Mb was used to define the region of interest around loci with significant *F*_ST_ and/or π.

### Regions of the Genome Under Selection

**Figure 2.**
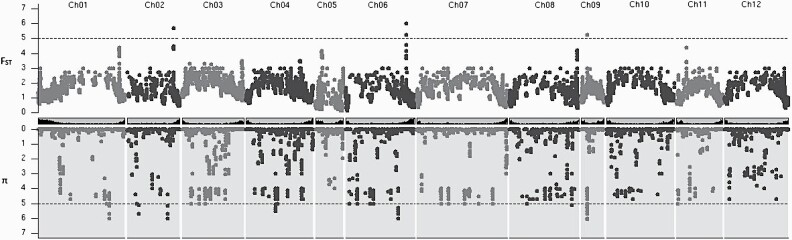
Chromosomal distributions of SNPs and outliers represented through Manhattan plots of −log_10_(*P*) for *F*_ST_ and π. Significant loci after Holm–Bonferroni correction are beyond the dotted line. Centre ideogram chromosomes show gene density. Plotted using KaryoploteR in R (Gel and Serra, 2017).

After combining significant SNPs within 3.93 Mb as potentially in LD and therefore marking the same region of the genome, 6 genomic regions with significantly (adjusted *P* < 0.05) low π in domesticates were identified (on chromosomes [chr] 1, 2 [2 regions], 4, 6, and 9) and 3 with significantly (adjusted *P* < 0.05) high *F*_ST_ were detected (on chr 2, 6, and 9; Table 2; Figure 2). The low π and high *F*_ST_ regions on chr 9 overlapped almost completely, whereas the others did not. There are other peaks in *F*_ST_, such as at the ends of chr 1, 3, and 8, and a highly heterogeneous distribution of π but these are not statistically significant. These 8 regions totaled ca. 63 Mb, approximately 5.6% of the eggplant genome and contained between 39 and 526 (mean 178.75) annotated genes. Of these, 375 had a significant (*e*-value < e^−10^) BLAST hit in Arabidopsis and 1113 had a significant BLAST hit in tomato (Supplementary Table 1). None of the putative orthologs had clear functions related to domestication traits in eggplant. In addition, we identified the genomic coordinates of molecular markers under the QTL peaks from the Doganlar et al. (2002) study and the prickliness indel marker from Miyatake et al. (2020) (Supplementary Table 2). In only one case was there overlap with a selective sweep; this was for the shoot anthocyanin QTL *sa6.1* which overlapped with sweep 6_2 on chr 6.

**Table 2.** Genomic regions of low diversity (low π in domesticated) or high differentiation (high *F*_ST_) in the comparison of the wild and domesticated eggplant populations

Region name	Test	*N* sig SNPs	Chr	Position	*N* genes
1_1	π	3	1	10 7964 200–11 5824 939	73
2_1	π	2	2	13 892 778–22 201 207	54
2_2	π	6	2	60 392 355–68 261 479	39
2_3	*F*_ST_	6	2	69 739 198–77 677 099	285
4_1	π	5	4	42 196 297–50 056 466	53
6_1	π	5	6	79 014 216–86 874 666	179
6_2	*F*_ST_	2	6	92 198 368–10 0135 870	526
9_1	π and *F*_ST_	16	9	5 546 492–13 498 317	221

### GO Analysis Reveals Putative Selection on the Photosynthetic Pathway

A GO analysis using the combined list genes in the 8 genomic regions putatively under selection was carried out, revealing significant (*P* < 0.05 after false discovery rate correction) over-representation for 171 GO terms (Supplementary Table 3). Many of the over-represented GO terms are related to photosynthesis, including *photosynthesis*, *photosystems I and II*, *chlorophyl* and *chloroplasts*, *thylakoids*, *plastids*, and *tetrapyrrole binding*. This included the putative orthologs of genes *PsbO-1* (*PS II oxygen-evolving complex*) and *LHCB5* (*light harvesting complex of photosystem II 5*), both of which are involved in photosystem II assembly (Jansson 1999; Popelkova and Yocum 2011).

Other terms involved in response to the environment including *response to osmotic stress* and *response to salt stress* as well as *response to hormone stimulus* were also uncovered.

## Discussion

In this study, measures of population differentiation between wild and domesticated populations of eggplants and nucleotide diversity within the domesticated population were used to identify genomic regions having the signature of selection during domestication.

Using our pipeline, identifying haplotype blocks in which outlier SNPs were found, we find that genomic regions putatively under selection during eggplant domestication were few in number and are each relatively small in size, ranging between 7.8 and 8.3 Mb and are found on 5 of the eggplant chromosomes (chr 1, 2, 4, 6, and 9). In total, this comprises about 5.6% of the eggplant genome; a similar analysis in tomato, but based on different methods to identify regions under selection, suggested about 1% of the genome was under strong selection (Sahu and Chattopadhyay 2017). That a small portion of the genome is shown to be under selection fits with the observation from QTL studies that in general a small number of QTL control domestication traits (Paterson 2002). For example, in maize, a small number of large effect genetic variants appear responsible for the transition from wild teosinte, whereas variants with smaller effects were responsible for improvement traits that contribute to standing variation in maize crops (Xue et al. 2016).

Eggplant and other members of the Solanaceae exhibit extensive chromosome-level synteny (Wu et al. 2009), so regions under selection can be compared. Indeed, it has already been reported that the QTL on chr 2 and 4 discussed above overlap with QTL for similar traits in tomato and pepper (Doganlar et al. 2002). Comparing our regions of selection to QTL maps for eggplant (Doganlar et al. 2002; Frary et al. 2003), it appears that regions on chr 2 and 4 may correspond to regions of the eggplant genome controlling fruit size and on chr 6 these regions may correspond to regions of the genome controlling prickliness and/or anthocyanin content. We attempted for test for overlap by looking for molecular markers closest to the QTL peaks in the Doganlar et al. (2002) study, and we did not find these markers in our selective sweeps (with one exception; see Results); however, sometimes the sweep and QTL region appeared close, and given that the QTL sometimes spanned 5–15 cM, there is possible overlap, even if the peaks do not coincide. We also note that the landraces are morphologically diverse and so we might not expect our comparison (wild vs. domesticated) to identify, for example, loci associated with fruit color or shape, given these are very variable in the landraces.

These regions collectively contained 1430 annotated genes, of which ca. 26% had a hit in the Arabidopsis genome and 78% had a hit in the tomato genome. GO enrichment analysis can reveal patterns in selection pressures and can be particularly useful when processing large lists of candidate genes. Using this, we found genes with orthologs related to photosynthesis were significantly over-represented in the regions showing a signal of selection. Photosynthesis is a pathway that has been identified as under selection in a number of crops including tomato (Koenig et al. 2013), other fruit crops (Cao et al. 2014; Li et al. 2019), and nonfruit crops (Pujol et al. 2008; Akakpo et al. 2017), and that has been suggested as a target for crop improvement (Long et al. 2015).

There is no consistent pattern in the change in photosynthetic rate in domesticated species relative to their wild relatives. Increase in photosynthetic rate was observed in domesticated rice (Cook and Evans 1983), soybean (Li et al. 2013), and cassava (Pujol et al. 2008), whereas photosynthetic rate and photosynthetic pigments were found to be reduced in domesticated yam relative to the wild progenitor (Padhan and Panda 2018). As domestication often involves a move from a nutrient poor to a nutrient rich habitat, an increase in photosynthetic rate can allow a domesticated species to grow more rapidly in a more protected and nutrient rich environment provided by human cultivation (Pujol et al. 2008). Photosynthetic capacity has been identified as one of the most important targets for increasing crop yield (Long et al. 2015) but has largely been under represented in crop improvement, perhaps due to the complexity of the genetics and the molecular mechanisms controlling traits related to photosynthesis (Mathan et al. 2016). As far as we are aware, photosynthetic rate has not been measured in wild and domesticated eggplant. Quantifying this will be an important next step to understanding the evolution of the photosynthetic pathway during domestication in eggplant.

It could also be that selection on genes related to photosynthesis results from selection on fruit ripening, a process in all fruit crops that involves several pathways that create appealing and edible fruit. In cultivated tomato, the presence of chloroplasts has been shown to be responsible for the green color in under ripe fruit (Cocaliadis et al. 2014). A distinguishing feature between wild and domesticated eggplants is that the fruit of wild eggplants retains its green color throughout ripening, until turning yellow when over ripe, while cultivated eggplants typically ripen to a characteristic purple (but also green, yellow, and white fruits are found, especially in landraces). Genes involved in chloroplast biosynthesis under selection during domestication may be responsible for fruit color change during ripening in eggplants as well as tomato.

Several other over-represented GO terms we identified related to traits which may have diverged during domestication, including a suite of terms related to response to the environment, including osmotic and salt stress, as well as terms potentially related to growth and vigor, including response to hormones and regulation of gene expression and metabolism. Furthermore, the subset of the genes without identifiable orthologs may be of importance to the domestication of eggplant, but we cannot ascribe any sort of function to them at this time. These could be targeted in the future using further in silico (Sadowski and Jones 2009, Galperin and Koonin 2014) or in vivo techniques (Alberts et al. 2015).

We were also limited in the scale of our analysis because a number of “wild” eggplants were previously identified as feral admixed individuals (Page, Gibson, et al. 2019). In the future, it will be important to expand our survey to include more true wild eggplants allowing a more robust analysis of the genomics of eggplant domestication.

## Conclusions

Sequencing the transcriptome is an effective way of reducing the complexity of the genome, making genome-scale analysis more computationally and cost efficient. However, this approach also comes with the caveat that only expressed genes will be sequenced; for example, in our recent work, we identified targets of selection on a gene-by-gene basis using transcriptome sequencing (Page, Gibson, et al. 2019), which would preclude the sequence analysis of nonexpressed or differentially expressed loci. In the present study, by mapping the expressed genes to the genome, we overcome this shortfall by identifying SNPs exhibiting signatures of selection (from transcriptome sequencing) and then interrogate the entire genomic region for candidate genes. Some of these signals of selection appear to overlap with previously identified domestication-related QTL, and we show that genes in these regions are enriched for functions related to photosynthesis, which is a relatively under-recognized and understudied domestication trait that has been linked to yield increase in cassava and fruit ripening in tomato.

## Supplementary Material

Supplementary data are available at *Journal of Heredity* online.

Supplementary Table 1. Gene names, taken from the eggplant genome gff files, and top Arabidopsis (A) and tomato (B) hits in the regions under selection.

Supplementary Table 2. QTL locations and potential overlap with selective sweep regions.

Supplementary Table 3. Over-represented GO terms in regions showing a signal of selection. P=biological process. F=molecular function, C=cellular component.

esab035_suppl_Supplementary_Table_1A

esab035_suppl_Supplementary_Table_1B

esab035_suppl_Supplementary_Table_2

esab035_suppl_Supplementary_Table_3

## Data Availability

RNA-seq data are available from the NCBI SRA under accession numbers SRR8736626–SRR8736638, SRR8736644–SRR8736648, SRR8736652, and SRR8736653.
